# Serum HBV Surface Antigen Positivity is Associated With Low Prevalence of Metabolic Syndrome in Korean Adult Men

**DOI:** 10.2188/jea.JE20140053

**Published:** 2015-01-05

**Authors:** Ja Sung Choi, Ki Jun Han, Sangheun Lee, Song Wook Chun, Dae Jung Kim, Hyeon Chang Kim, Hee Man Kim

**Affiliations:** 1 Department of Internal Medicine, Myongji Hospital, Goyang, Republic of Korea; 2 Department of Internal Medicine, Catholic Kwandong University College of Medicine, Gangneung, Republic of Korea; 3 Department of Internal Medicine, Ajou University School of Medicine, Suwon, Republic of Korea; 4 Department of Preventive Medicine, Yonsei University College of Medicine, Seoul, Republic of Korea; 5 Department of Internal Medicine, Yonsei University Wonju College of Medicine, Wonju, Republic of Korea

**Keywords:** chronic hepatitis B, metabolic syndrome, survey, triglycerides

## Abstract

**Background:**

Metabolic syndrome has clinical implications for chronic liver disease, but the relationship between chronic hepatitis B and metabolic syndrome remains unclear. The aim of this study was to determine whether hepatitis B surface antigen (HBsAg) positivity is associated with metabolic syndrome.

**Methods:**

Data were obtained from the Third Korean National Health and Nutrition Examination Survey (KNHANES). Participant sera were tested for HBsAg. Metabolic syndrome was defined according to the modified National Cholesterol Education Program Adult Treatment Panel III guidelines for Koreans.

**Results:**

Of the 5108 participants, 209 (4.1%) tested positive for HBsAg, and 1364 (26.7%) were diagnosed with metabolic syndrome. The prevalence of metabolic syndrome was 23.4% in HBsAg-positive men, 31.5% in HBsAg-negative men, 18.6% in HBsAg-positive women, and 23.7% in HBsAg-negative women. After adjusting for multiple factors, male participants who tested positive for serum HBsAg had an odds ratio of 0.612 (95% confidence interval [CI] 0.375–0.998) for metabolic syndrome and an odds ratio of 0.631 (95% CI 0.404–0.986) for elevated triglycerides. Women who tested positive for serum HBsAg had an odds ratio of 0.343 (95% CI 0.170–0.693) for elevated triglycerides.

**Conclusions:**

Positive results for serum HBsAg are inversely associated with metabolic syndrome in men and with elevated triglycerides in men and women. This suggests that elevated triglycerides may contribute to the inverse association between HBsAg and metabolic syndrome.

## INTRODUCTION

Chronic hepatitis B, which results from hepatitis B virus (HBV) infection, is widespread in Korea. In 2009, the prevalence of hepatitis B surface antigen (HBsAg), a marker of HBV infection, was 3.2% in Korea.^1^ Chronic HBV infection can lead to the development of liver cirrhosis and/or hepatocellular carcinoma.^2^ The majority of chronic HBV infections in Korea are acquired in the perinatal period, though some HBV infections are acquired via horizontal transmission.

Metabolic syndrome, also known as insulin resistance syndrome, is a constellation of metabolic disorders, including increased waist circumference, hyperglycemia, elevated blood pressure, and an abnormal lipid profile. In Korea, the prevalence of metabolic syndrome has increased markedly in recent years, from 24.9% in 1998 to 31.3% in 2007.^3^ Metabolic syndrome is associated with liver diseases, including non-alcoholic fatty liver disease, chronic hepatitis C virus (HCV) infection, and hepatocellular carcinoma. Non-alcoholic fatty liver disease is a hepatic manifestation of metabolic syndrome.^4^ In chronic hepatitis C, metabolic syndrome is a potential independent predictor of liver-related mortality.^5^ Insulin resistance reduces the response to anti-HCV treatment.^6^ Metabolic syndrome is a possible risk factor for hepatocellular carcinoma, independent of the hepatitis virus.^4^^,^^7^ However, to the best of our knowledge, there are few existing studies on the association between metabolic syndrome and chronic hepatitis B. Three studies conducted in Asian populations reported that chronic hepatitis B is associated with a low prevalence of metabolic syndrome.^8^^–^^10^ However, the study populations were limited to small geographic areas, which may have led to selection bias. In addition, the statistical models used in these three studies were not adjusted for all potentially confounding factors.

The Third Korean National Health and Nutrition Examination Survey (KNHANES III) is a population-based examination survey conducted in 2005 by the Korean Centers for Disease Control and Prevention and the Korean Ministry of Health and Welfare.^11^ KNHANES III was designed to yield data representative of the Korean population. Thus, use of KNHANES III data will hopefully prevent selection bias that may have been unavoidable in the aforementioned studies focused on smaller geographic areas.^8^^–^^10^

HBV is endemic to Korea, and the prevalence of metabolic syndrome is also increasing in Korea.^1^^,^^3^ The aim of this study was to use representative data from KNHANES III to investigate the possible association between HBsAg and metabolic syndrome and to determine if there are correlations between HBsAg and components of metabolic syndrome, including elevated blood pressure, elevated blood glucose, elevated triglycerides, low high-density lipoprotein (HDL) cholesterol, or increased waist circumference.

## METHODS

### Study population and data

The KNHANES III data used in this study included anthropometric measurements, questionnaire responses, and blood chemistry tests. Enrolled participants were ≥20 years of age and underwent an examination including a test for serum HBsAg, which was detected using an electrochemiluminescence immunoassay. A total of 5108 participants were selected and their data analyzed. Approval of this study was obtained from the Institutional Review Board of Myongji Hospital, Goyang, Korea, complying with the Treaty of Helsinki.

### Parameters and definitions

Metabolic syndrome was defined based on the modified criteria for Koreans from the National Cholesterol Education Program Adult Treatment Panel III.^12^ Participants were categorized into three groups by Korean districts according to the population: large city, small-medium city, or rural area. Subjects with a body mass index ≥25 kg/m^2^ were considered obese. Increased waist circumference was defined as a waist circumference ≥90 cm in Korean men and ≥85 cm in Korean women.^13^ Subjects were considered to have hypertension when systolic blood pressure was ≥140 mm Hg, diastolic blood pressure was ≥90 mm Hg, or they had a medical history of antihypertensive drug use. Type 2 diabetes mellitus was defined as a fasting blood glucose ≥126 mg/dL or a medical history of hypoglycemia or insulin use. Subjects who had smoked more than 100 cigarettes in their life were categorized as smokers, while current alcohol consumption was defined as consumption of more than one glass of alcohol in the last year. Planned, systematic, and repeated physical activity to improve or maintain physical strength was considered regular exercise. Income status was classified into five categories: <1 000 000 Korean Won/month, ≥1 000 000 and <2 000 000, ≥2 000 000 and <3 000 000, ≥3 000 000 and <4 000 000, and ≥4 000 000. Education level was classified into five categories: none (did not complete elementary school), only through elementary school, only through middle school, only through high school, and through college.

### Statistical analysis

Chi-square tests and *t*-tests were used to assess differences in anthropometric features, laboratory test results, personal medical history, and health behaviors between participants with and without chronic hepatitis B. Logistic regression analysis was used to investigate the association between chronic hepatitis B and metabolic syndrome. Logistic regression analysis was also used to investigate the associations between chronic hepatitis B and the five components of metabolic syndrome, including elevated triglycerides, elevated blood pressure, elevated blood glucose, low HDL cholesterol, and increased waist circumference. SPSS software version 11.0 (SPSS, Inc., Chicago, IL, USA) was used for analysis. *P*-values <0.05 were considered statistically significant.

## RESULTS

### Baseline characteristics of participants

A total of 5108 participants were analyzed, consisting of 2144 men and 2964 women (Table 1). Mean ± standard deviation age was 47.1 ± 15.1 years. The prevalences of HBsAg positivity and metabolic syndrome were 4.1% and 26.7%, respectively. Of the 107 men participants who were HBsAg-positive, 23.4% also had metabolic syndrome. Of the 2037 men participants who were HBsAg-negative, 31.5% had metabolic syndrome. There was no significant difference in the prevalence of metabolic syndrome between HBsAg-positive and HBsAg-negative men (*P* = 0.077). Of the five components of metabolic syndrome, only prevalence of elevated triglycerides was significantly lower in HBsAg-positive men than in HBsAg-negative men (29.0% *vs.* 38.5%, *P* = 0.047). Of the 102 woman participants who were HBsAg-positive, 18.6% had metabolic syndrome. Of the 2862 woman participants who were HBsAg-negative, 23.7% had metabolic syndrome. There was no significant difference in the prevalence of metabolic syndrome between HBsAg-positive and HBsAg-negative women (*P* = 0.233). Of the five components of metabolic syndrome, only prevalence of elevated triglycerides was significantly lower in HBsAg-positive women than in HBsAg-negative women (8.8% *vs.* 20.7%, *P* = 0.003).

**Table 1.** Baseline characteristics of all participants (*n* = 5108)

Variable	All (*n* = 5108)	Men			Women		
	
		HBsAg Positive (*n* = 107)	HBsAg Negative (*n* = 2037)	*P*	HBsAg Positive (*n* = 102)	HBsAg Negative (*n* = 2862)	*P*
Age (year)^a^	47.1 (15.1)	45.4 (12.5)	47.4 (14.6)	0.112	46.6 (13.5)	46.9 (15.5)	0.855
Gender (men), *n* (%)	2144 (42.0%)	—	—		—	—	
Location (large city), *n* (%)	2201 (43.1%)	42 (39.3%)	696 (34.2%)	0.313	34 (33.3%)	963 (33.6%)	0.710

BMI (kg/m^2^)^a^	23.7 (3.3)	24.2 (3.0)	24.0 (3.1)	0.466	24.0 (3.7)	23.5 (3.4)	0.170
Systolic pressure (mm Hg)^a^	119.1 (17.8)	119.6 (13.0)	123.0 (16.2)	0.011	118.7 (19.6)	116.3 (18.5)	0.201
Diastolic pressure (mm Hg)^a^	77.3 (10.7)	79.2 (9.5)	80.9 (10.4)	0.106	75.7 (10.1)	74.7 (10.3)	0.328
Waist circumference (cm)^a^	81.0 (9.7)	84.6 (8.4)	84.3 (8.8)	0.790	79.5 (9.3)	78.5 (9.6)	0.263

AST (IU/L)^b^	22.0 [19.0–27.0]	27.0 [22.0–39.0]	24.0 [20.0–29.0]	0.000^d^	23.0 [20.8–29.0]	20.0 [17.0–24.0]	<0.0001^d^
ALT (IU/L)^b^	18.0 [13.0–25.0]	30.0 [21.0–47.0]	22.0 [17.0–31.0]	<0.0001^d^	18.0 [15.0–23.3]	15.0 [12.0–20.0]	<0.0001^d^
Fasting blood glucose (mg/dL)^b^	90.0 [85.0–98.0]	91.0 [85.0–99.0]	92.0 [86.5–101.0]	0.331^d^	88.0 [83.0–96.3]	89.0 [84.0–96.0]	0.600^d^
Total cholesterol (mg/dL)^b^	183.0 [160.0–206.0]	179.0 [153.0–199.0]	184.0 [161.0–206.0]	0.050^d^	181.5 [156.0–201.3]	182.0 [160.0–206.0]	0.320^d^
HDL cholesterol (mg/dL)^a^	45.1 (10.8)	43.2 (10.3)	42.2 (10.1)	0.337	47.9 (10.6)	47.1 (10.9)	0.443
Triglyceride (mg/dL)^b^	105.0 [75.0–157.0]	106.0 [76.0–158.0]	126.0 [88.0–188.5]	0.011^d^	84.5 [65.0–109.3]	94.0 [68.0–136.0]	0.014^d^

Metabolic syndrome, *n* (%)	1364 (26.7%)	25 (23.4%)	641 (31.5%)	0.077	19 (18.6%)	679 (23.7%)	0.233
Elevated triglyceride, *n* (%)	1418 (27.8%)	31 (29.0%)	785 (38.5%)	0.047	9 (8.8%)	593 (20.7%)	0.003
Elevated blood pressure, *n* (%)	1860 (36.4%)	44 (41.4%)	938 (46.0%)	0.319	30 (29.4%)	848 (29.6%)	0.962
Elevated blood glucose, *n* (%)	1130 (22.1%)	25 (23.4%)	577 (28.3%)	0.266	16 (15.7%)	512 (17.9%)	0.568
Low HDL cholesterol, *n* (%)	2875 (56.3%)	48 (44.9%)	933 (45.8%)	0.849	63 (61.8%)	1831 (64.0%)	0.648
Abdominal obesity, *n* (%)	1306 (25.6%)	29 (27.1%)	539 (26.5%)	0.883	26 (25.5%)	712 (24.9%)	0.888
Hypertension, *n* (%)	1361 (26.6%)	30 (28.0%)	638 (31.3%)	0.475	23 (22.5%)	670 (23.4%)	0.840
Diabetes mellitus, *n* (%)	437 (8.6%)	9 (8.4%)	225 (11.0%)	0.394	7 (6.9%)	196 (6.8%)	0.995
Obesity^c^, *n* (%)	1665 (32.6%)	43 (40.2%)	728 (35.7%)	0.350	33 (32.4%)	861 (30.1%)	0.624

Smoking, *n* (%)	1934 (37.9%)	83 (77.6%)	1636 (80.3%)	0.488	3 (2.9%)	212 (7.4%)	0.087
Alcohol, *n* (%)	3287 (74.9%)	83 (77.6%)	1743 (85.6%)	0.023	63 (61.8%)	1938 (67.7%)	0.207
Exercise, *n* (%)	2432 (47.6%)	56 (52.3%)	1051 (51.6%)	0.881	43 (42.2%)	1282 (44.8%)	0.599
Income status, *n* (%)	*n* = 5022	*n* = 103	*n* = 1999	0.315	*n* = 102	*n* = 2818	0.336
<1 000 000 Won	1269 (25.3%)	17 (16.5%)	472 (23.6%)		20 (19.6%)	760 (27.0%)	
≥1 000 000 but <2 000 000 Won	1462 (29.1%)	34 (33.0%)	605 (30.3%)		36 (35.3%)	787 (27.0%)	
≥2 000 000 but <3 000 000 Won	1196 (23.8%)	23 (22.3%)	480 (24.0%)		22 (21.6%)	671 (23.8%)	
≥3 000 000 but <4 000 000 Won	517 (10.3%)	16 (15.5%)	213 (10.7%)		10 (9.8%)	278 (9.9%)	
≥4 000 000 Won	578 (11.5%)	13 (12.6%)	229 (11.5%)		14 (13.7%)	322 (11.4%)	
Education level, *n* (%)	*n* = 5065	*n* = 107	*n* = 2018	0.089	99	2841	0.004
none	500 (9.9%)	2 (1.9%)	88 (4.4%)		11 (11.1%)	399 (14.0%)	
only through elementary school	776 (15.3%)	6 (5.6%)	268 (13.3%)		15 (15.2%)	487 (17.1%)	
only through middle school	590 (11.6%)	15 (14.0%)	239 (11.8%)		23 (23.2%)	313 (11.0%)	
only through high school	1891 (37.3%)	43 (40.2%)	789 (39.1%)		35 (35.4%)	1024 (36.0%)	
through college	1308 (25.8%)	41 (38.3%)	634 (31.4%)		15 (15.2%)	618 (21.8%)	

ALT, alanine aminotransferase; AST, aspartate aminotransferase.

^a^Data are expressed as mean (SD).

^b^Data are expressed as median [25–75 percentiles].

^c^Obesity was defined as body mass index ≥25 kg/m^2^.

^d^*P* values were obtained using Mann-Whitney U test for skewed variables: AST, ALT, fasting glucose, total cholesterol, and triglyceride.

### Serum HBsAg and metabolic syndrome

In a multivariate analysis, the odds ratio of a positive result for HBsAg was 0.612 (95% confidence interval [CI] 0.375–0.998) in men with metabolic syndrome and 0.695 (95% CI 0.400–1.208) in women with metabolic syndrome, after adjusting for age, location, smoking habits, alcohol consumption, exercise habits, income status, and education level (Table 2).

**Table 2.** Multivariate analysis of the association between positive HBsAg and metabolic syndrome

Model	Men (*n* = 2144)			Women (*n* = 2964)		
	
	Odds ratio	95% CI	*P* value	Odds ratio	95% CI	*P* value
Unadjusted	0.664	0.420–1.049	0.079	0.736	0.444–1.221	0.235
Model 1^a^	0.659	0.412–1.054	0.082	0.721	0.416–1.249	0.243
Model 2^b^	0.656	0.410–1.049	0.079	0.715	0.413–1.239	0.232
Model 3^c^	0.612	0.375–0.998	0.049	0.695	0.400–1.208	0.197

CI, confidence interval.

^a^Adjusted for age.

^b^Adjusted for age, location and exercise habits.

^c^Adjusted for age, location, smoking habits, alcohol consumption, exercise habits, income status, and education levels.

Multivariable analyses were performed to explore the associations between a positive result for HBsAg and the five components of metabolic syndrome. The odds ratio for elevated triglycerides was 0.631 in HBsAg-positive men (95% CI 0.404–0.986) and 0.343 in HBsAg-positive women (95% CI 0.170–0.693; Table 3). Elevated triglycerides were inversely associated with HBsAg positivity.

**Table 3.** Multivariate analysis of the association between positive HBsAg and metabolic abnormalities

	Dependent variables				

	Elevated triglyceride	Elevated blood pressure	Elevated blood glucose	Low HDL cholesterol	Increased waist circumference
Men (*n* = 2144)					

Positive HBsAg^a^					
Odds ratio	0.631	0.856	0.803	0.915	1.042
95% CI	0.404–0.986	0.558–1.314	0.491–1.314	0.608–1.376	0.660–1.645
*P* value	0.043	0.477	0.382	0.670	0.860

Women (*n* = 2964)					

Positive HBsAg^a^					
Odds ratio	0.343	1.037	0.840	0.862	1.014
95% CI	0.170–0.693	0.623–1.725	0.476–1.483	0.569–1.305	0.624–1.648
*P* value	0.003	0.890	0.548	0.482	0.955

CI, confidence interval.

^a^Adjusted for age, location, smoking habits, alcohol consumption, exercise habits, income status, and education level.

## DISCUSSION

Our study shows that serum HBsAg positivity is inversely correlated with prevalence of metabolic syndrome in Korean men but not in women. This is consistent with findings from other Asian studies.^8^^–^^10^ A Taiwanese study reported that the prevalence of metabolic syndrome was 8.0% in HBsAg-positive subjects and 10.9% in HBsAg-negative subjects.^8^ Another study performed in China reported that the prevalence of metabolic syndrome was 5.9% in HBsAg-positive participants and 8.8% in HBsAg-negative participants.^10^ In both of these studies, this inverse association remained even after adjusting for age and sex. A study performed in Hong Kong reported that the prevalence of metabolic syndrome was 11.0% in patients with chronic hepatitis B and 20.2% in controls without chronic hepatitis B.^9^ However, these three studies did not stratify subjects by sex, as our study did. The definition of metabolic syndrome differs according to sex, and analysis should be performed separately for men and women.^12^^,^^13^ Our study conducted separate analysis according to sex and found that only HBsAg-positive males had a lower prevalence of metabolic syndrome than their HBsAg-negative counterparts. In addition, our study used a nationally representative population, while the other studies did not.

In the present study, a positive result for serum HBsAg was associated with a low prevalence of elevated triglycerides. This low prevalence might be an important contributor to the inverse association between HBsAg positivity and metabolic syndrome, which would be consistent with findings in other studies.^9^ Chen et al. reported that HBsAg positivity is associated with lower prevalence of hypertriglyceridemia,^14^ while Su et al. reported that chronic hepatitis B is associated with low serum levels of triglycerides.^15^ However, the exact mechanism causing the low levels of triglycerides in HBsAg-positive patients remains unclear. One study suggested that the hepatitis B virus X protein is a contributor to low serum triglycerides.^16^ The role of HBV in lipid metabolism should be further explored.

Metabolic syndrome is related to liver fibrosis. A cross-sectional study in Taiwan reported that metabolic syndrome increases the risk of liver cirrhosis in patients with chronic hepatitis B.^17^ Metabolic syndrome has been linked to increased risk and severity of liver fibrosis through activation of hepatic stellate cells.^18^ However, liver cirrhosis itself can cause metabolic disturbances.^19^ These observations suggest a relationship between HBV infection and diabetes mellitus; development of diabetes mellitus may contribute to secondary changes due to HBV infection but not to HBV infection itself.^20^ In addition, hepatic fibrosis in an HBV-infected liver is caused by virus-induced liver injury rather than insulin resistance.^21^ Thus, the cause and effect relationship between metabolic syndrome and liver cirrhosis in patients with chronic hepatitis B remains unclear.^22^

Chronic hepatitis C is associated with insulin resistance, but the association between chronic hepatitis B and insulin resistance is not well-established. Huang et al. reported that chronic hepatitis B without cirrhosis is not associated with type 2 diabetes mellitus.^20^ Kumar et al. reported that chronic hepatitis B is not correlated with insulin resistance.^23^ On the other hand, a study using NHANES III data reported that type 2 diabetes mellitus and insulin resistance are independent predictors of overall mortality in chronic hepatitis B, although only 66 participants in the study had chronic hepatitis B.^5^

Our study had several limitations. First, various conditions associated with HBsAg were not considered because the KNHANES tested only for the presence of HBsAg and hepatitis B surface antibody. An individual who is HBsAg-positive may be a healthy carrier, have chronic active hepatitis, or have liver cirrhosis. In terms of serology, HBsAg positivity is associated with various levels of HBV DNA concentration and two states of hepatitis B e antigen. Further stratification of HBsAg states should be performed to determine the exact role of HBsAg in the development of metabolic syndrome.

Second, the KNHANES did not differentiate between liver cirrhosis and chronic hepatitis. Liver cirrhosis is closely related to glucose intolerance, regardless of its cause.^19^ Diabetes may develop as a complication of cirrhosis, which is called hepatogenous diabetes.^24^ Therefore, liver cirrhosis may have contributed to the metabolic derangement observed in our study. In addition, two recent studies reported conflicting results on the association between metabolic syndrome and liver cirrhosis in chronic hepatitis B patients.^17^^,^^21^ One of these studies reported that metabolic syndrome increases the risk of liver cirrhosis in chronic hepatitis B patients.^17^ However, the other reported that hepatic fibrosis in HBV-infected liver is caused by virus-induced liver injury, rather than by insulin resistance.^21^ The data suggest that metabolic syndrome is not a risk factor for liver cirrhosis but an outcome of liver cirrhosis.^21^ Therefore, liver cirrhosis must be excluded when clarifying the effect of HBsAg on metabolic syndrome.

Third, our study was a cross-sectional study, and therefore it was not possible to explore a causal relationship between chronic HBV infection and metabolic syndrome. It is unclear whether chronic HBV infection suppresses development of metabolic syndrome. In addition, horizontal transmission could not be completely excluded from subjects with chronic infection resulting from vertical transmission; in this situation, chronic hepatitis B infection could have been followed by development of metabolic syndrome.

In conclusion, data from KNHANES III indicate that HBsAg positivity is associated with a low prevalence of metabolic syndrome in men, and this association may be partly attributed to low prevalence of elevated triglycerides.
